# Internet of Things in Pregnancy Care Coordination and Management: A Systematic Review

**DOI:** 10.3390/s23239367

**Published:** 2023-11-23

**Authors:** Mohammad Mobarak Hossain, Mohammod Abul Kashem, Md. Monirul Islam, Md. Sahidullah, Sumona Hoque Mumu, Jia Uddin, Daniel Gavilanes Aray, Isabel de la Torre Diez, Imran Ashraf, Md Abdus Samad

**Affiliations:** 1 Department of Computer Science and Engineering, Dhaka University of Engineering and Technology (DUET), Gazipur 1707, Bangladesh; mobarak3112@gmail.com (M.M.H.); drkashemll@duet.ac.bd (M.A.K.); 2 Department of Software Engineering, Daffodil International University, Daffodil Smart City (DSC), Birulia, Savar, Dhaka 1216, Bangladesh; monirul.swe@diu.edu.bd; 3 Department of Computer Science and Engineering, Asian University of Bangladesh (AUB), Bangabandhu Road, Tongabari Ashulia, Dhaka 1349, Bangladesh; 4 School of Kinesiology, University of Louisiana at Lafayette, Lafayette, LA 70504, USA; 5 AI and Big Data Department, Endicott College, Woosong University, Daejeon 34606, Republic of Korea; jia.uddin@wsu.ac.kr; 6 Higher Polytechnic School, Universidad Europea del Atlántico, Isabel Torres 21, 39011 Santander, Spain; 7 Universidad Internacional Iberoamericana, Campeche 24560, Mexico; 8 Universidade Internacional do Cuanza, Cuito EN250, Bié, Angola; 9 Department of Signal Theory, Communications and Telematics Engineering, Unviersity of Valladolid, Paseo de Belén, 15, 47011 Valladolid, Spain; 10 Department of Information and Communication Engineering, Yeungnam University, Gyeongsan 38541, Republic of Korea

**Keywords:** patient monitoring, Internet of Things, healthcare in IoT, m-health, maternal care coordination, maternal health data-set, risk factor, medication reminder, prenatal care, neonatal care, postpartum care, antenatal care

## Abstract

The Internet of Things (IoT) has positioned itself globally as a dominant force in the technology sector. IoT, a technology based on interconnected devices, has found applications in various research areas, including healthcare. Embedded devices and wearable technologies powered by IoT have been shown to be effective in patient monitoring and management systems, with a particular focus on pregnant women. This study provides a comprehensive systematic review of the literature on IoT architectures, systems, models and devices used to monitor and manage complications during pregnancy, postpartum and neonatal care. The study identifies emerging research trends and highlights existing research challenges and gaps, offering insights to improve the well-being of pregnant women at a critical moment in their lives. The literature review and discussions presented here serve as valuable resources for stakeholders in this field and pave the way for new and effective paradigms. Additionally, we outline a future research scope discussion for the benefit of researchers and healthcare professionals.

## 1. Introduction

The Internet of Things (IoT) plays a pivotal role in remote health care and monitoring to increase medical devices’ efficacy, velocity and accessibility [1,2]. IoT collects remote patient health data using wearable sensors and devices connected to an internet-based health monitoring system [3,4]. The IoT has been a primary driver of globalization and has promoted the convergence of electronic communications and media services that can be leveraged to monitor the patient’s condition from anywhere for successive feasibility of all associated stakeholders [5,6,7].

Regular pregnancy monitoring is unquestionably essential for the eventual development of an infant from embryo to full-fledged human being [8]. The cost surge contributed to the installation of such devices, primarily on hospital premises. However, the emerging need for a personalized care model [9,10] is becoming evident over time. In this context, “its” refers to the healthcare system under discussion. Pregnant women are expected to endure factors such as environmental conditions, traffic, transportation options, mobility and overall accessibility to obtain regular monitoring from these devices. This system and its associated devices usually consist of user data acquisition components, a central server and remote doctors who serve as expert stakeholders in this ecosystem [11,12].

IoT devices are often used for care management tasks related to women during and after pregnancy. Care coordination in primary care involves deliberately organizing patient care activities and sharing information between all participants and stakeholders involved in patient care to achieve safer and more effective care [6]. Remote patient monitoring is one of the most common applications of IoT devices in healthcare. This approach addresses various challenges in healthcare by leveraging IoT devices. For example, IoT devices can automatically collect a wide range of health metrics, including heart rate, blood pressure and temperature, from patients who are not physically present in a healthcare facility. This capability eliminates the need for patients to travel to healthcare providers or collect these data themselves [13,14]. Continuous and automatic monitoring of patients offered by IoT devices plays a crucial role in improving healthcare care delivery. Examples include products such as glucose monitoring devices that effectively eliminate the need to keep records manually. These devices can alert patients if glucose levels reach a critical stage [6,15]. IoT devices are effective in monitoring pregnant women [16]. Feasibility studies are carried out to understand to what extent pregnant women can be helped. Machine learning, IoT and wearables can effectively monitor the health and safety of pregnant women at home and in hospitals [13,16].

Previous research in the field of IoT technology and its applications in healthcare, patient monitoring and pregnancy monitoring has exhibited several limitations, including data security and privacy in their IoT health monitoring equipment, confronting issues in sensor accuracy and facing the continual issue of maintaining security across all layers of IoT, from perception to application layers, to handle increasing security threats [2,3,4,5,6,7]. There is a lack of comprehensive systematic reviews in these domains, with previous studies failing to synthesize existing knowledge effectively. Specifically, previous research has inadequately explored the role of IoT in pregnancy use cases and there is a scarcity of usability and user experience research in the literature. Additionally, the discussion of interoperable IoT protocols in healthcare technology often lacks specific details.

### 1.1. Research Gaps

A systematic dive into related review papers related to the topic provided a substantial gap in this area that motivated us to provide a systematic literature study that would help all practitioners, stakeholders and experts in this domain. The gaps we have identified were:

1. A few pieces of literature adequately discussed IoT and patient monitoring systems. However, a significant inadequacy related to the role of IoT was observed in pregnancy use cases.
2. Although there were some contributions to the body of knowledge through review articles, no systematic reviews of the literature were observed.
3. In the reviews, a complete summary of usability, issues, human-computer interaction and user experience research was absent.

### 1.2. Contributions

The gaps mentioned above in the research motivated us to provide a systematic review of the literature that would be extensive for all stakeholders, contribute a large amount to the body of knowledge and help stakeholders in advancing the field. Specific contributions are highlighted in the following:

- We have provided extensive related work in this domain that identifies the research gap that would effectually enrich the body of knowledge in IoT and pregnant women’s research domain.
- It provides a systematic analysis of accumulated works on the role of IoT in the monitoring and management of pregnant women. We have followed the PRISMA guideline, a standardized guideline and identified 42 notable research articles in this domain. The research field is currently witnessing prominent trends, with both IoT and pregnant women garnering substantial attention.
- We have outlined the leading role of IoT, which is continuous monitoring and management and explored devices, architectures, systems and studies that are compatible with the care of pregnant women.
- The literature and related works helped us to understand the challenges in adoption, which we have outlined separately, followed by a comprehensive dissection through discussions and implications for future works that would help researchers in this domain advance the field of research.

In this study, we conducted a systematic review of the literature on state-of-the-art IoT-based systems, applications and research output that assist pregnant women through pregnancy to neonatal care. The systematic review of the literature includes an in-depth and comprehensive analysis of various relevant work on monitoring methods, previous surveys and reviews of the literature on pregnancy monitoring in Section 2 and a comprehensive review of research methods in Section 3. The detailed review of the literature is presented in Section 4, followed by a conclusion that identifies the challenges, issues, opportunities, implications and future study in this domain.

## 2. Related Work

**Figure 1:**
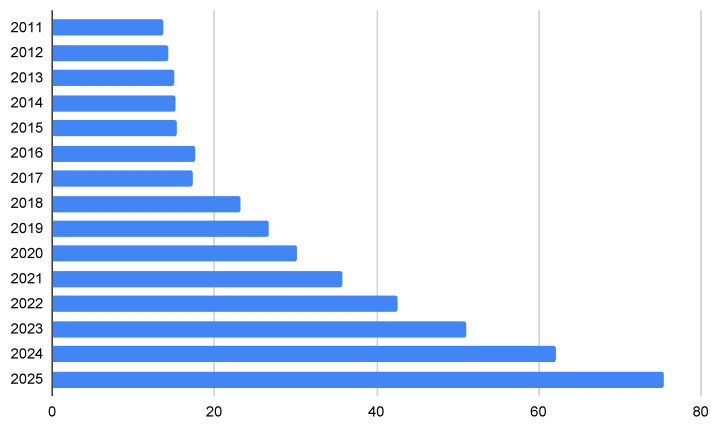
Growth of number of connected IoT devices by year (in-billions).

IoT technology has effectively contributed to a massive paradigm shift in all sectors of the world that resonated and reverberated with the amalgamation of information and technology [17,18,19,20]. IoT devices are expected to increase to a staggering 75 billion by 2025, according to data generated by [21], drawn in Figure 1. Several research works in this field have contributed to the massive development of the IoT and its applications to a greater extent [22]. In terms of pregnancy monitoring, a plethora of research initiatives and review articles related to IoT in the medical field, patient monitoring and pregnancy monitoring are available, which, upon further discussion in the following subsection, would be the building blocks of our literary contribution through systematic literature work.

### 2.1. IoT and Healthcare

IoT in healthcare is used primarily with applications and services. The main application areas of IoT include medication reminders and management. In contrast, services cover a wide range of use cases [23]. Several review papers are available in the IoT and Healthcare domain. The authors of Kashani et al. [24] conducted important work covering a comprehensive systematic overview that includes applications, techniques and trends in IoT for the healthcare domain. Selvaraj and Sundaravaradhan [25] worked on pointing out the challenges and opportunities that are present in IoT-based healthcare systems through a detailed review process that generously helped the body of knowledge in this domain. Panchatcharam and Vivekanandan [26] focused on reviewing smart health, monitoring and surveillance management, as well as IoT architectures along with security analysis for data transfer in IoT devices in the healthcare domain. Javaid and his team provided essential insights on IoT’s usage in the ensuing COVID pandemic [27] and Bharadwaj et al. [28] investigated the roles machine learning can play while being embedded in IoT devices and wearables to generate actionable insights. An innovative IoT healthcare solution is presented, introducing an intelligent medication box designed to support the elderly and people with chronic diseases in the management of a variety of medications. This system comprises six specialized sub-boxes, timely reminders of the mobile app and biometric sensors to monitor temperature and heart rate, improving adherence to medication for a wide spectrum of medical needs [29]. In addition, the study addresses the issue of non-compliance with medications through IoT-based solutions, offering an adaptable platform for pharmaceutical packaging, wearable sensor-based vital parameter monitoring and user-friendly interfaces to improve patient care. While specific medications are not detailed, the system caters to a diverse range of medical needs [30]. Another IoT-based reminder system is introduced, aimed at patients with dementia, but versatile enough to benefit all users by ensuring timely medication through notifications and employing an IoT-enabled Arduino device with infrared sensors to monitor medication adherence, regardless of the type of medication [31]. Furthermore, an IoT-based smart medication dispensing system is presented, enhancing medication adherence for various medical conditions and minimizing healthcare service delays by ensuring timely and unscheduled medication based on real-time patient vitals [32]. An RFID-based IoT system is also introduced for medication control, promoting medication management for different medical needs, particularly in the context of Ambient Assisted Living for Older People [33]. The versatile application of IoT extends to medication management, encompassing a wide spectrum of patient requirements [23]. Services in this field cater to various use cases, underscoring the adaptability of IoT in the effective management of medications. In [34], the authors explore the latest developments in telemedicine, contributing to disease prevention mechanisms and expanding the scope of healthcare services through IoT technologies [34].

### 2.2. IoT and Patient Monitoring

**Figure 2:**
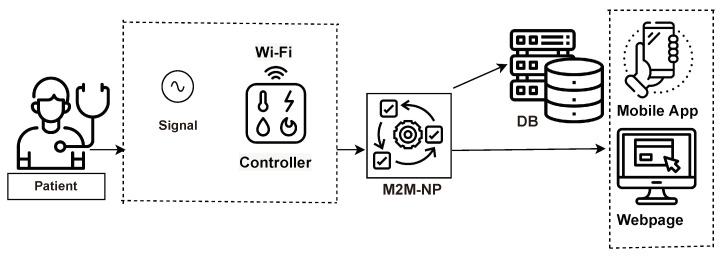
Architecture of an IoT based patient monitoring system.

Several research works have been proposed on the role of IoT in patient monitoring systems where the knowledge domains included architecture, application, services and patient management [35,36]. Figure 2 provides an overview of architecture provided in [35] that gives us an idea of the workflow of IoT-based patient monitoring systems, a general overview of its construction and how the IoT devices are embedded in the system.

Several review papers were contributed to the body of knowledge to strengthen the advancement of knowledge in this domain [36]. The formal work includes a review of the study in [37] where the authors investigated cloud computing and web services in combination with IoT and its subsequent role in patient monitoring activities. Rajan Jeyaraj and Nadar [38] interestingly appended IoT to add deep learning in the knowledge domain to understand the impact deep learning has on patient monitoring systems. El-Rashidy et al. [39] provided a comprehensive review of the role of mobile-assisted devices and chronic diseases in health monitoring by highlighting the principles, trends and challenges associated with each study. Similar studies were carried out in [40] where the authors provided a review of electronic health systems for the management of diabetes. The authors of [41] pointed out the role of Raspberry PI in IoT and subsequent usage in health monitoring systems. Wearable technologies and their implications for IoT health monitoring are investigated in [42]. Finally, Coulby et al. [43] summarized the accessibility of IoT technology by pointing out the current state-of-the-art and providing actionable insights from experimental design.

Several studies have explored the implementation of IoT devices for maternal and newborn care. Singh et al. [44] utilized deep machine learning on a Softlayer-based cloud computing infrastructure to integrate neonatology intensive care units for real-time vital sign recording. Gadekar and Vaze [45] developed a logistic regression model for a Neonatal Health Monitoring System with alarm capabilities. Singh et al. [46] designed unique data transmission protocols and communication interfaces for NEO, enabling real-time data transmission and automated vital sign monitoring. El-Aziz and Taloba El-Aziz and Taloba [47] utilized Arduino UNO microcontrollers to monitor neonatal incubators, focusing on vital signs such as pulse rate, temperature, humidity and gastrointestinal air. de Oliveira Filho et al. [48] implemented an IR non-invasive monitoring system to capture thermal images and monitor temperature in incubators. Kalilani et al. [49] introduced the Blynk IoT platform for newborn incubators, which offers body temperature measurement, ambient temperature control and humidity control. Kshirsgar et al. [50] used the DHT11 framework to control temperature and humidity in neonatal incubators. Nivetha and Kumar [51] employed microcontrollers and sensors for intensive care incubators, monitoring heartbeat data and using cry alarms. Cay et al. [52] introduced signal processing algorithms and MQTT-IoT-based networking for NeoWear, focusing on breathing rate and movement tracking. De et al. [53] incorporated MCC and WSN SMCC to monitor newborn body temperature, movement tracking and heart rate. Shakunthala et al. [54] combined IoT and sensor modules in incubators to provide SMS services, alarms and sensor-based monitoring. Cay et al. [55] employed MQTT networking architecture and signal processing algorithms for IMU-Baby-Guard, using pressure sensor pads to monitor newborns. Govindaraj et al. [56] utilized IoT and sensor modules for incubator monitoring systems, including alarm systems and newborn baby monitoring. Lenka et al. [57] integrated cloud, IoT and sensor modules into neonate monitoring systems to collect temperature, heart rate, crying sound and moisture data to determine newborn needs and coordination of care.

### 2.3. IoT and Pregnancy Monitoring

IoT and pregnancy monitoring are surveyed through basically two-pointers. The first is monitoring methods, applications and use cases; the other is medication and reminder management [34]. The work mainly involves the prenatal, neonatal, prepartum and postpartum phases of pregnancy that monitor both stakeholders, namely mothers and babies [58,59,60,61].

Ahmed et al. [16] reviewed and analyzed the risk factors associated with maternal health and investigated the usefulness of IoT in this regard concerning remote areas. Albahri et al. [34] discussed state of the art in telemedicine that helps in disease prevention mechanisms. Ettiyan et al. [58] performed a comprehensive survey on the health monitoring of pregnant women and the role of IoT in these systems. Similar but updated research was carried out in [62] that surveyed the exact role of IoT up to 2022.

Our comprehensive investigation of previous studies concluded that, although some of the review articles [16,58,59,60,61,62,63] address IoT and its subsequent role in pregnancy monitoring methods and systems, very few systematic reviews of the literature have been performed to date in this domain that summarize the research that is ultimately shown in Table 1. The penultimate goal of the systematic review has always been to identify, evaluate and summarize the results, evaluation and findings of all relevant studies systematically on an issue, which is health monitoring for pregnant women in this case, thus making the available evidence more accessible and usable to decision makers and relevant stakeholders. We have organized the paper with separate subsections for IoT and Healthcare and IoT and Patient Monitoring to facilitate a clear and logical flow of information. In the section ‘IoT and Healthcare’, we introduce the research gap, which, along with the limited availability of review work on pregnant women, provided us with substantial motivation to carry out the systematic review of the literature, as discussed in the subsequent section.

In our comprehensive study, we have addressed the shortcomings discovered in various existing survey articles on pregnant women’s health monitoring systems. These limitations included various areas, including the absence of technical specifics, the lack of clarity and framework, concerns about data security and privacy and the oversight of external contextual factors. By acknowledging and discussing these limitations, we aimed to provide a comprehensive and critical assessment of the current state of research in this field, encouraging future work to address these issues and advance the development of more effective and context-sensitive health monitoring systems for pregnant women.

### 2.4. Standardization of IoT Protocols for Healthcare Systems

A crucial aspect of IoT in healthcare care is interoperability, which facilitates seamless data sharing and communication between diverse systems and devices. In this review, we examine the issues and improvements around interoperable IoT protocols in the context of healthcare technology. We recognize the importance of interoperability issues in IoT health technology and the necessity for standardization, despite the fact that the precise names of protocols are not mentioned in the examined publications. The adoption of interoperable protocols is crucial to ensuring effective data sharing, security and connection within IoT-based healthcare systems, according to several studies.

In IoT-based healthcare systems, Panchatcharam and Vivekanandan [26] emphasized the need for consistent data transfer and security analyses. Similarly to this, Javaid and Khan [27] talked about how IoT can help with healthcare care issues, especially during the COVID-19 pandemic, which called for interoperable systems for effective data exchange and monitoring. In a review of the role of machine learning in allowing healthcare applications based on the IoT, Bharadwaj et al. [28] emphasized the importance of interoperability for data-driven healthcare solutions. To demonstrate the usefulness of interoperable healthcare care solutions, Yew et al. [35] proposed an IoT-based real-time remote patient monitoring system. Rahaman et al. [36] study of the evolution of the IoT-based smart health monitoring systems also touched on the need for data sharing and interoperability of the system. Interoperable protocols are essential to ensure the connectivity of healthcare devices and services, as demonstrated by Mohammed et al. [37] to investigate remote patient monitoring using web services and cloud computing. A patient monitoring system for IoT-based healthcare was discussed by Rajan Jeyaraj and Nadar [38], who noted that interoperable protocols for thorough data sharing could be advantageous. The principles, trends and difficulties of interoperability in healthcare technology were also highlighted in the study [39] of mobile health for remote patient monitoring in chronic diseases.

Even though the evaluated papers do not go into detail about the specific protocols, these studies collectively highlight the crucial role that interoperable IoT protocols play in overcoming obstacles and developing healthcare technologies. In order to achieve effective data transfer, security and connectivity in IoT-based healthcare systems, which ultimately benefit patients and healthcare providers, standard protocols are essential.

## 3. Materials and Methods

In this article, we have followed a systematic method of conducting IoT and its role in the health monitoring of pregnant women through a comprehensive literature study. To our knowledge, we have presented the role of the subject in various stages of pregnancy through an objective analysis of all the principles related to the subject. We summarized the main points after going through all the related articles published in this section.

### 3.1. Search Criteria

**Figure 3:**
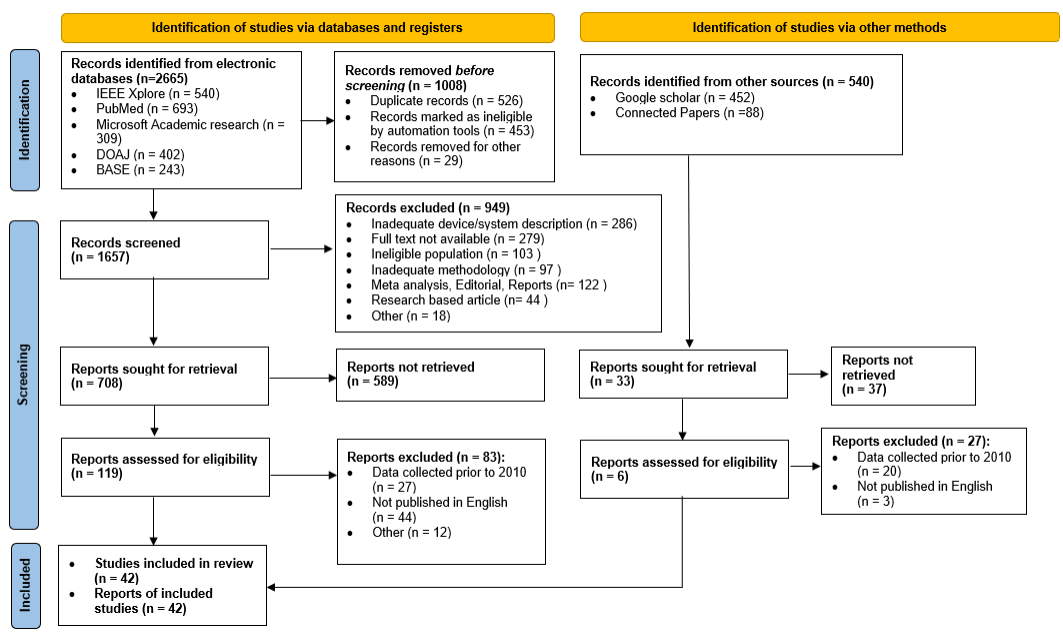
PRISMA flow diagram for the systematic review in this research.

We started our research following the PRISMA guideline [64]. We searched for articles using specific keywords related to the role of IoT in the health monitoring of pregnant women. Academic search engines such as IEEE Xplore, Google Scholar, Microsoft Academic Research, Directory of Open Access Journals (DOAJ) and Bielefeld Academic Search Engine (BASE) were used as sources to search for all related papers. We coupled that with a website named Connected Papers (https://www.connectedpapers.com, accessed on 10 August 2023) that generated a node graph of connected papers to examine the interlinkage between the papers in detail. The keywords we used to search for articles included ‘technology and maternal health’, ‘use of the IoT in patient monitoring’, ‘use of the IoT in pregnant women’, ‘IoT technologies for prenatal care’, ‘IoT and antenatal care’, ‘IoT and postpartum care’, ‘Care coordination of pregnant women through technology’, ‘technology in neonatal care’, ‘wearable technologies for pregnant women’, ‘embedded devices for pregnant women’, ‘medication management tools for pregnant women’, ‘smart devices for pregnant care’, ‘remote maternal health monitoring’, ‘expectant care coordination through IoT’, ‘IoT architectures for pregnant women’ and ‘IoT-based systems for pregnant women’, as well as ‘antenatal’, ‘prepartum’ and ‘antepartum’. Figure 3 illustrates the PRISMA flow diagram for the inclusion and exclusion of articles for the systematic review in this research.

### 3.2. Eligibility Criteria

We have followed the PRISMA guidelines [64] for the review work. Systematically searching through different journals, conferences, workshops and symposiums using the search method mentioned above, we have found 383 papers, projects and reports on IoT and its role in the health monitoring of pregnant women.

After reviewing the relevance of studies, record identification, significance and pertinence screening and deleting duplicate works, we selected 42 articles. However, to understand the evolution of IoT and its significance in patient monitoring and, specifically, pregnancy management, more research was added to the related work and challenges section, which ultimately provided us with research gaps, challenges and opportunities in this domain. The selection and filtering process is summarized as follows:

- The research must have discussed IoT devices, architectures, or systems for monitoring or managing pregnant women’s healthcare conditions through the prenatal and neonatal phases.
- A clear methodology must have been presented regarding the devices used or architectures discussed in the studies.
- Unique, relevant, important, significant and informative works are included.
- Relevant review articles were included in related works to identify the research gap.
- Duplicate research works were excluded.

## 4. Results and Discussion

This section dives deeply into the systematically selected literature that contributes to the IoT in the pregnancy domain. This section provides a brief overview of the main contribution of IoT, which is monitoring and evaluation and describes the stages of pregnancy and where IoT has been used. In addition to exploring the contribution, the literature review also discusses the limitations that researchers encountered through a subsequent comparative analysis. Furthermore, the section engages in a thorough exploration of the limitations encountered by researchers, as identified through a comparative analysis of the literature. This analysis addresses the research questions about adopting IoT devices and the challenges faced in implementing IoT systems in maternal healthcare.

### 4.1. IoT Services in Pregnancy Monitoring

IoT has provided tremendous services in pregnancy monitoring. Before we dive into the literary works on this issue, it is important to point out the stages of pregnancy that this review initiative has considered within its scope. We have examined two stages of pregnancy, namely prenatal care and neonatal care. Therefore, the words antenatal, prepartum and antepartum are synonyms of prenatal care for this study and postnatal and postpartum fall under the jurisdiction of neonatal care. In recent years, IoT technology has played a pivotal role in maternal healthcare. Studies by Shermi et al. [65], Sarhaddi et al. [66], Liu et al. [67] and Ahmed and Kashem [68] have explored various aspects of IoT applications in maternal health monitoring. Ahmed and Kashem [68] introduced an IoT-based risk level prediction model for maternal healthcare in the context of Bangladesh. Although these studies collectively showcase the diverse applications and potential of IoT to improve maternal health monitoring and care, it is important for future research to dive into quantitative assessments to provide specific data and statistics on the efficiency and effectiveness of applying IoT in the coordination and management of care during pregnancy. This will offer a more comprehensive understanding of the benefits and outcomes associated with the use of IoT in pregnancy care.

Table 2 provides an overview of the literature that discusses prenatal care and the role of IoT, while Table 3 dives into the extensive role of IoT in monitoring methods, applications and characteristics of neonatal care that were developed through research proposals carried out by researchers. The following two subsections describe specifically IoT’s role in prenatal and neonatal care for the pregnancy monitoring domain.

We found an app that monitors fetal growth, which is crucial to prenatal treatment. This app tracks fetal movements, temperature, heartbeat and labor discomfort. The device uses various sensors and IoT technology such as accelerometers, force sensors, GSM, microcontrollers, pulse rate sensors, sweat sensors and temperature sensors [65,69]. The comprehensive monitoring provided by this application offers several advantages, including more convenient and efficient routine fetal monitoring. However, it is important to acknowledge that the system’s accuracy in real-world scenarios may be influenced by potential threats to external validity, such as false labor pain or variations in environmental conditions, like hot weather sweating [72]. Addressing these challenges and ensuring the system’s reliability in diverse situations are critical areas for further research and development.

#### 4.1.1. IoT in Prenatal Care

Priyanka et al. [63], developed a smartphone application to automatically track a pregnant woman’s health and the fetus’ activity. They used Global System for Mobile Communication (GSM) modules coupled with Arduino and associated IoT technologies that could detect and report abnormal traits such as diabetes rate, pressure value and temperature in pregnant women. The author claimed that the system was able to collect associated data with the aid of Arduino Uno and IoT technology and they were able to transfer the collected data to a mobile application. In this developed system, GSM transmission was used to send an alert message as soon as any abnormality was detected. They used IoT technology with accelerometer sensors, heartbeat sensors, temperature sensors, blood pressure sensors, real-time BP sensors, Arduino UnO and WiFi modules for accurate results. The mentioned system works well in real-time and is quite effective. Although this study proposed an IoT-based solution for real-time monitoring of pregnancy vitals using GSM modules, the validity, accuracy and cost-benefit analysis of the proposed system and the protection of sensitive health data were not described.

Shermi et al. [65] developed an application that can be used to monitor the movements, temperature and heartbeat of the fetus. In addition, it has been used to monitor signs of labor discomfort, such as heavy sweating and light contractions. This would make it much easier to perform routine fetal monitoring. They utilized an Accelerometer sensor, Force sensor, Global System for Mobile (GSM), Microcontroller, Pulse rate sensor, Sweat sensor, Temperature sensor and IoT technology. This will help a lot more to get the mother to the hospital on time. This study did not discuss how the device will maintain accuracy in the presence of potential threats to the external validity of the proposed system, such as false labor pain and sweating from hot weather.

Sarhaddi et al. [66] presented an Internet-of-Things (IoT)-based system in this article to offer widespread maternal health monitoring throughout the pregnancy and postpartum stages. Several data collectors in the system monitor the condition of a mother, including those that monitor stress, sleep and physical activity. The discussion of integrating the presented system with the current healthcare system concludes with the sending of a self-reported questionnaire to participants using the cross-platform application to collect their background data. The questionnaire is designed to gather information about their diagnosed illnesses, previous miscarriage or preterm birth, way of life and level of perceived stress. The investigation has limited generalizability primarily because it does not include a comparison between traditional care and health outcomes. Therefore, it is challenging to draw broad conclusions or apply the findings to a wider population.

Haliima et al. [69], developed a non-intrusive Internet-of-Things (IoT) and data analytics architecture for the cloud to help women find addiction therapy while pregnant. The device will monitor, collect and evaluate crucial data from pregnant women to spot emergencies using sensors built into a wristwatch. The system automatically contacts the required services in an emergency and transfers the processed data to the cloud for storage. Furthermore, as shown in previous works, using data analytics can provide greater insight and help make the right decision. They have used lightweight IoT-based technology and the Wireless Body Area Networks (WBAN) layer, the Fog Computing Layer, the Medical Service Layer and the Cloud Computing Layer in their system. The study does not provide information on the reliability and validity of the survey questionnaire, which can affect the quality of the data collected. Additionally, questions remain about its suitability and applicability for rural areas with limited access to electricity, the Internet and technical support.

Li et al. [70], developed a cloud computing platform and the IoT based on wearable technology to provide intelligent maternal healthcare services. Pregnant women are the focus of the Internet-based Smart Maternal platform, which can significantly reduce the workload of medical staff, increase productivity, facilitate prenatal visits to the doctor and raise the level of obstetric care. The network layer, the application layer and the service layer make up most of the platforms designed in this paper. They then assisted in the analysis of the data collected from the questionnaire using the SPSS statistical program. However, the study is subject to potential data accuracy challenges due to factors such as physical conditions, compliance and data monitoring regulations.

Ahmed and Kashem [68], developed a system to efficiently monitor and forecast the 279 level of risk for pregnant women in the context of Bangladesh. The level of intensity of risk will be assessed using health data and risk factors for pregnant mothers using this system. This work aims to estimate the risk level based on pregnancy risk indicators using appropriate analytical tools and machine learning algorithms. A dataset was created entirely from scratch. They also used the Modified Decision Tree Algorithm, demonstrating an accuracy score of 97%. The study merits its use of multi-source data collection, risk groups-based analysis and the accuracy of the proposed endeavor. However, internal validation (survey questionnaire) and external validation (representativeness of the sample population) of the predictive model would improve the robustness of the findings in the study.

Venkatasubramanian [71] developed an automated system that would allow medical professionals to use IoT to forecast and control the monitoring of Maternal and Fetal Health (MFH). Using IoT sensors, data-based feature extraction and an intelligent system based on the Deep Convolutional Generative Adversarial Network (DCGAN) classifier, this research offers a method to monitor high-risk MHF. Numerous clinical signs are continuously tracked, including the mother’s uterine tone, blood pressure, oxygen saturation and heart rate of the Maternal-Fetal (MF) monitoring system. The IoT algorithm and a generative adversarial network model architecture, along with data collected from gadgets and sensors, were used in their system. From gadgets and sensors, they collected data. Poor patients would find it challenging to use the system due to the high cost of the layers, sensors and other components used to build the system. However, the generalizability of the findings is not clear due to the absence of a broader study sample or diverse settings. Additionally, ethical concerns regarding data collection further impact the applicability of the results beyond the specific research context.

Gopalakrishnan et al. [72], proposed an IoT-based method to track the vital signs of a pregnant woman and her fetus. At a sociosystemic level, the solution ensures that pertinent pregnant people, doctors, midwives, the government and neighborhood NGOs are informed of crucial information (stakeholders). They created a tool that relieves stress, keeps track of a pregnant woman’s vital signs individually and establishes systemic connections between the pregnant woman and healthcare stakeholders. They combined IoT-based algorithms with systems-based intervention, brainstormed concepts and mappings algorithms to obtain an accurate result. The electronic parts cost approximately 1500 BDT (USD 15). It is costly in the context of a poor country and poor people. Furthermore, the lack of comparative analysis and the risk of confidentiality breaches relating to sensitive data can be considered a weak study factor.

Santhi et al. [73], created a wearable device that continuously logs data while monitoring the critical parameters that need to be tracked for a patient. If a patient encounters a life-threatening situation, this device will raise the alarm and use the CC3200’s built-in WiFi to connect to the web app. The product is small and wearable; it will gather and transfer information to the doctor as soon as possible. For accurate results, they used a C4.5 Decision Tree classification algorithm with blood pressure, heart rate, temperature sensors, IoT, CC3200 and IoT-based technology. Setting up a sensor on a device and carrying both a device and sensor pose challenges for patients with limited means, as sensors and other components are expensive [79]. Two limitations we have observed in their study are related to validation and the analysis of applicability in a real-world setting.

Megalingam et al. [74], described the integrated healthcare system, which includes ultrasound screening and monitoring vital parameters, especially for pregnant women living in remote areas. The need to measure vital parameters in expectant women, the technique they created for this and the experimental setup were thoroughly discussed. To store the parameters that the healthcare worker measured, they used a Secure Digital (SD) card. All prenatal problems are collected and sent to the hospital on this SD card. They built their solution using an IoT-based algorithm and a few components (DS 1620 IC, PQRS waveframe and non-invasive technology). It is challenging to obtain precise ultrasound scanning results and deliver data wirelessly to an SD card. One of the major limitations identified in this study is the lack of discussion of this large volume of data processing mechanisms. Furthermore, the low sample size also raises the question of generalizability, thus eventually affecting the accuracy of the data and the applicability of the system in real-world setups.

Sato et al. [75], suggested a compact and flexible sensor system that can be safely connected to the mother’s abdomen and capture the electrocardiogram (ECG) signal. To make easy prenatal status monitoring possible at home, the authors proposed a system that consists of a mechanism that the sensor wirelessly transmits the fetal heart rate (FHR) and the maternal heart rate (MHR) to the mother’s smartphone. They created a flexible sensor with a patch-like design that is only 72 mm by 38 mm by 7 mm (19.2 cm^3^) and weighs 13 g. This sensor can be connected wirelessly and securely to smartphones using Bluetooth Smart. The FHR and MHR are extracted using an MCU built into the sensor to save power. They used IoT technology. They had a wireless sensor network, an FHR sensor, a CR2032 primary battery, an ECG waveform and a 19.2 cm^3^ flexible sensor. The technology and components make it difficult to calculate MHR and FHR rates and sensors are pricey for the less fortunate, which we note as one of the general limitations of the study.

Lyu et al. [76], suggested an Android OS-based multicommunication fusion-based mobile vital signs monitoring system for expectant women. Patients can keep an eye on maternal and fetal data wherever they choose. They can also receive comprehensive care by sending data to the server for additional processing and remote diagnosis. Furthermore, the accuracy of remote diagnosis has increased. A multi-communications Fusion-Base user terminal was employed. The mobile terminal and the monitoring sensors are the two physical parts of the terminal. The monitoring sensors are linked to a tablet PC through wired (serial port, USB, etc.) or wireless (Bluetooth, Zigbee, RF433, etc.) data paths. They used a technique called wavelet analysis. This system needs to implement some crucial techniques, including structure, features and algorithms, for better results. The system is based on multi-communication fusion, which relies highly on a good internet connection, making it unsuitable for resource-constrained areas. Furthermore, the study does not provide any data on the performance of the proposed system, such as its precision, reliability and user satisfaction.

Saarikko et al. [77] performed an evaluation regarding IoT and wearable. The specific objective of this study was to evaluate the viability of using a smart wristband and an IoT-based monitoring system to continuously monitor the health parameters (physical activity, sleep and heart rate) of women throughout pregnancy and until one month after giving birth. The smart bracelet using IoT technology was a viable approach to gathering representative data on continuous variables of health parameters during pregnancy. Continuous monitoring gives information in real-time in between consultations, which may help with a patch-like personalized prenatal follow-up. A cloud server was connected to the smart wristband. However, the study is limited by the feasibility of an observational design, subject to confounders. We also note the use of convenience sampling and selection bias in their study that eventually led to low statistical power, which may also be attributed to a smaller sample size. Validation, particularly with respect to certain aspects of the study, was also necessary and is considered lacking to some extent in the study.

Anudeep et al. [78] focused on promoting the most critical protection measures by effectively communicating the benefits of vaccines and other medications. This approach helped reduce the mortality rates for infants and pregnant women. They also proposed a solution that involves affordable monitoring devices with robust data security. These devices collect essential information from relevant individuals, reducing the need for patients to travel long distances for medical visits. To implement their system, they used a cloud infrastructure that integrates with the IoT and employed the RSA (Rivest-Shamir-Adleman) algorithm. The study did not provide details on the survey methodology, such as the response rate and sampling methods, which could have affected the external validity. Furthermore, in their study, a lack of analysis of the reasons for reluctance to use health care is observed. There may be constraints other than timely communication from the hospital end, reported in their study, that require further correlation and constraint analysis with different variables.

#### 4.1.2. IoT in Neonatal Care

Singh et al. [44], developed a neonatal intensive care unit (iNICU). This system reduces the time required to provide clinical care by integrating several essential calculators for neonatal care, including the dextrose, calories and nutrition calculators. They used deep machine learning (DML), HTML5, PostgreSQL and JSON-based REST APIs, associated with IoT technologies that could perform continuous monitoring, data recording and notifications related to every newborn that were easily accessible through the cloud-based application. The study does not provide information on the effectiveness of the iNICU solution in clinical outcomes or scalability in resource-limited settings. Furthermore, limited validation to prevent incorrect data entry is observed in their study with limited clinical evidence, which eventually poses the risk of potential interpretation bias.

Gadekar and Vaze [45] reviewed existing health monitoring systems and emphasized the major challenges associated with these systems. They talked about various medical applications and their advantages while paying particular attention to systems for tracking one’s health. The architecture and foundational technologies for IoT-based neonatal health monitoring were also covered in the article. This review paper dictates the limitations of existing studies regarding IoT device-based monitoring of neonates, including lack of non-obstructive surveillance that maintains a natural environment for fragile babies, poor reliability and quality of service for high-risk patients, low power consumption, transmission delay, node failure and network communication.

Singh et al. [46], developed a device called “NEO” that consists of three modules: a data acquisition module, a data transmission module and a display interface module, that was able to gather vital information in real-time by integrating with various devices and sensors connected to newborns in neonatal intensive care units (NICU). However, the confidentiality risk is an issue that was absent from their discussion, along with high integration costs and infrastructural challenges.

El-Aziz and Taloba [47] developed a neonatal incubator device that provides babies with optimum temperature, relative humidity, optimum light and an appropriate level of oxygen, the same as those in the womb. This system consists of an Arduino UNO microcontroller and several sensors associated with IoT technologies that can detect temperature and humidity. A comparative analysis of efficacy and cost integration was expected from the researcher’s end, which we note as one of the limitations of the study.

de Oliveira Filho et al. [48], developed a risk management system for neonatal incubators that can detect temperature anomalies in the newborn to prevent or quickly respond to risk situations like hypothermia and hyperthermia. The system used Flir’s Lepton long wave infrared (LWIR) array sensor as its main part to collect 160×120 temperature readings from the neonatal incubator. The study only focuses on temperature monitoring and does not consider other vital signs that could be important in neonatal care, which we note as one of the limitations of the study.

Kalilani et al. [49] proposed a neonatal incubator unit that could provide a low-cost neonatal incubator. They used the Blynk IOT platform associated with several sensors for their device. The system includes a heart rate and blood oxygen saturation detection system that alerts users to abnormal heart rate and blood oxygen readings via an application based on a predetermined expected range. We note that potential ethical considerations were absent from the study. Furthermore, the study does not provide information on the long-term effectiveness or safety of the incubator unit developed.

Kshirsgar et al. [50] suggested a design for a baby incubator that would be practical to use in a rural setting as a business location. Using this system, they successfully developed a reasonable, portable and life-saving infant incubator system. For temperature control and sensing, they used a Raspberry Pi in conjunction with a DHT11 sensor, considering the mobility problem, a common occurrence in rural areas. The sensors’ physiological data collection may not be as accurate as comparable medical grade equipment. The solution depends on the patient constantly having a smartphone with them, which might not be possible. Patients with restricted mobility or cognitive disabilities who might have trouble using the smartphone application may not be a good fit for the system. The lack of information in the research on the implementation costs of the suggested system may be a drawback for healthcare providers with limited resources.

Nivetha and Kumar [51], in their work, extensively discussed pediatric and neonatal intensive care units that can provide pediatric monitoring and infant ICU. They used several sensors and parameters associated with the MSP430 microcontroller for their proposed system. The temperature rate, the cry alarm and the humidity rate were the main features of this device. However, our findings suggest skepticism, as they do not include a cost-benefit analysis and require discussion related to resource-constrained areas.

Cay et al. [52], developed a smart textile chest band called “NeoWear” that can track physiological parameters and breathing rate. They used signal processing algorithms associated with the MQTT-based IoT networking architecture for their system. Movement monitoring, pressure tracking and breathing count are the main features of their system. We find three points to report on this research: (a) controlled environment: high-fidelity programmable NICU baby mannequin used in the study may not be representative, (b) clarity in safety: safety and comfort of NeoWear system is not clear and (c) representative analysis: Lack of comparative cost-benefit analysis, which in total represents the limitation of the study.

De et al. [53], utilized sensor mobile cloud computing (SMCC) to develop a neonatal health monitoring system with a wireless sensor network (WSN) and mobile cloud computing (MCC) as building blocks. Neonatal body temperature, acceleration caused by movement and heart rate are tracked in the authors’ system using temperature, acceleration and heart rate measurement sensors. The sensor data is kept in the cloud. The health professional uses a mobile Android application for newborn monitoring to continuously monitor and obtain these data with a dedicated alarm system. However, privacy and security concerns were not addressed in the study, which remains a limitation of their findings.

Shakunthala et al. [54] suggested a technique with characteristics that include temperature, humidity, heart rate of the newborn, gas leakage and light intensity in the incubator that are continuously monitored using the IoT. The doctor or the chosen physician will receive an alarm message by email or SMS if these metrics go beyond the threshold level. This method can continuously update the parameters in the cloud and reduces the need for a person to monitor newborns regularly. However, privacy and security concerns were not addressed in the study, which remains a limitation of their research.

Cay et al. [55], designed and developed the smart “Baby-Guard” textile chest band, which uses IoT to monitor respiratory rates and detect apnea. A sensor belt, a wearable embedded system and an edge computing device make up the neonatal wearable system known as the Baby Guard. An Inertial Measurement Unit and textile-based pressure sensors make up the sensor belt (IMU). A microprocessor with wireless communication and power management makes up the wearable system. The wearable system and the edge computing device (ECD) are linked through an MQTT networking architecture. However, the research lacks controlled testing and evaluation of the proposed system and scalability issues.

Govindaraj et al. [56] proposed an alarm system integrated with IoT architectures and systems to alert stakeholders in the event of an unexpected condition of newborns. They informed that as newborns cannot control their temperatures, automated incubators are gaining momentum to solve the problems and their approach is an IoT-enabled alarm system to alert health professionals and stakeholders in emergency situations by monitoring biological parameters for multiple incubators. However, we report a data security issue, which was expected from their end, as medical data remains very sensitive in nature.

Lenka et al. [57] proposed an IoT enabled, cloud-integrated and sensor-inclusive architecture for monitoring neonate activities by collecting heart rate, temperature data, crying sound data and moisture data to determine the needs of neonates and care coordination. Apart from the unaddressed potential ethical concern, whether the variables used for the architecture are representative or not remains a valid question without the validation of expert personnel in the field.

### 4.2. IoT Services in Pregnancy Medication Management

The IoT also provides substantial services in managing the condition of expectants in their time of need. From medication reminders to alarms to stress-free activity management that enables pregnant women toward seamless delivery and postdelivery care coordination. In our study, we justify the merit of a separate section related to management rather than merging with monitoring because while monitoring provides continuous care coordination, discreet management merits a separate discussion of the role of IoT in pregnancy medication management that is outlined in this section through the literature that focused primarily on management and reminder through IoT services.

Bjelica et al. [80] provides a concept of an IT ecosystem for prenatal care based on the fusion of various services in an e-health ecosystem that has been semantically enhanced. Research also explored usability, user experiences and the technology acceptance model (TAM) to understand the applicability and acceptance of such an ecosystem. However, we note the limitation in validation and reliability, as the validity and reliability of the measurement questionnaire are not described and remain subject to bias or social desirability effects due to self-reported data.

Oti et al. [81], deployed an IoT-enabled management device and system that uses a real-time heart rate-based k-means algorithm to assess stress levels and report them for further management tasks by associated stakeholders. Their study included a case study on maternal health, in which 20 pregnant women were remotely observed for 6 months of pregnancy and 1 month after delivery, where the main function of this system was data collection. We observe that the study does not discuss any control group and has a minimal sample size, thus limiting generalizability. Furthermore, the indicators of stress as evaluation parameters remain limited in their study.

Moreira et al. [82] proposed using averaged one-dependence estimators, a machine learning approach, to interpret real-time pregnancy data from IoT devices and gateways. This statistical method helped decentralize data preprocessing and intermediate storage, minimizing the quantity of data that must be sent to the cloud and ensuring operability even in a network failure. This algorithm can identify high-risk circumstances for expectant mothers with hypertensive disorders of pregnancy, which can result in serious problems, including death for both the expectant mother and the fetus. They used a dataset that could recognize a patient’s condition and trigger a warning for more care. Researchers are expected to discuss ethics and privacy as a form of concern from all stakeholders, whose absence we note as a limitation in their study.

Jara et al. [83] proposed a system based on the IoT for drug identification and medication monitoring. Radio Frequency Identification (RFID) and Near Field Communication (NFC) were the main features of their system. However, researchers are expected to debate ethical issues and privacy as a concern for all parties. Furthermore, while suitable for initial design and modeling, the devices require full-scale investigation into reproducible scalability.

Eswari and Priya [84] also worked on drug identification mechanisms but differed in characteristics in which they also offered the detection of health anomalies from vital signs. They opined that drug compliance and adverse drug reactions (ADRs) are two of the most important issues in the healthcare arena related to patient safety. However, in the study, no empirical evidence of the effectiveness and feasibility of the proposed system in improving drug compliance and reducing ADR is seen in the study.

Beri et al. [85], proposed a framework for intelligent health monitoring IoT and fog-assisted monitoring that can obtain and process the parameters of the temperature, blood pressure, ECG and pulse oximeter of a pregnant woman. They used real-time series data and rule-based algorithms associated with fog computing to analyze their system. Privacy and ethical issues are important issues that were expected from the authors, as the data remain very sensitive in nature.

Reddy et al. [86] developed a remote-controlled device that can provide management to pregnant women. They used several sensors associated with IoT-based technologies for accurate measurements that had pressure tracking, data collection, data storage, temperature tracking, heartbeat condition and SMS service as the main features of their system. However, it remains unclear to us what the validity and reliability of the instrument are. Furthermore, potential challenges like false alarms and ethical concerns with real-time analysis were expected to be discussed at their end, which we note as a major limitation of their study.

Alotaibi et al. [87] proposed a smart mobile pregnancy system to promote pregnancy awareness and remote management. They used the smartphone platform and the IoT technology. The system aims to empower expectant mothers with enough information and pertinent knowledge about pregnancy and engage them in more physical activity using cutting-edge technology, particularly in remote areas. The research lacks information on the sample size and characteristics of pregnant women who participated in testing the system. In addition, potential barriers or challenges that may arise in the implementation and adoption of the proposed social networking technology and platform, such as user engagement, privacy concerns and cultural factors, were absent from their report.

Chen et al. [88], developed an IoT application to manage obstetric outpatient information and prenatal genetic testing requirements. They used an IoT platform associated with existing medical information systems for complete medical image detection. Several stakeholders, including researchers in this domain and especially mothers, would benefit from the actionable insights generated from the image data developed through the IoT application for healthcare management. Research is limited by effectiveness constraints and feasibility issues in subsequent adoption.

Ghimire et al. [89] devised and developed a non-invasive, straightforward and affordable IoT monitoring and management system as part of an architectural framework for prenatal care. They employed an IoT-based architecture, which acts as a key system for self-imperative care in routine prenatal screening tests at the convenience of the home. The research has limited generalizability and has eminent data security concerns as medical data remains very sensitive and merits subsequent discussion from researchers on handling those.

Musyoka et al. [90] developed a smartwatch that can monitor ambulatory blood pressure and blood pressure readings for the expectant mother 24 h a day. An alert is sent to the assigned caregiver to initiate immediate action in an emergency. They used a rapid prototyping approach associated with IoT-based and cloud-based applications for their system that was evaluated with 30 expectant mothers from Kenyan hospitals. The study’s small sample size limits its potential to be generalized and the fact that medical data are still extremely sensitive makes data security a serious problem. As a result, researchers should consider how to handle such data in the next research.

Ashu and Sharma [91] discussed the characterization, processing and use of big data in healthcare. They proposed a telemedicine-induced approach to manage the fetal condition of the expectant. For monitoring, they used wearable monitors and IoT devices that generated fetal data, which was later fed into machine learning approaches to obtain a classification result on fetal health. However, one drawback is that security must be considered when working with such massive amounts of data. Although there may not always be sufficient data available for accurate conclusions from analysis on a specific dataset, this constraint may also be related to the accuracy or dependability of big data predictions.

Kumar et al. [92] suggested an automated system that employs wearable technology and fuzzy neural techniques to detect postpartum hemorrhage (PPH). The tools assess the temperature, pulse rate, blood pressure and frequency of sweating of the pregnant woman and warn doctors about severe bleeding in expectant mothers during delivery. Due to the small sample size, unclear accuracy of the suggested system and lack of a cost-benefit analysis, the generalizability of the research is constrained.

Niela-Vilén et al. [93] used IoT and smartwatch technologies to track pregnant women to analyze differences in stress, physical activity and sleep. This study looked at daily well-being trends in pregnant women both before and after COVID-19 pandemic-related nationwide stay-at-home bans. The researchers reported that mild changes in stress, physical activity and sleep were consistent with a more prolonged pregnancy. IoT technologies are being used to track daily well-being patterns for pregnant women. Due to the small sample size, the generalizability of the investigation is limited. The study is based on self-reported data, which may be erroneous or biased. It has possible confounding effects, which we flag as a study limitation.

### 4.3. Discussion on the Reviewed Literature

The study put forward by us provides an extensive overview of the IoT’s role in prenatal and neonatal care. According to our observation, in terms of prenatal care, IoT played an extensive role in managing, monitoring, reminding and reporting expectant activities to stakeholders, thus generating actionable insights. In neonatal care, an extensive role is seen in intelligent incubator-based research where various sensors, IoT technologies, devices and applications were designed to coordinate neonates’ care. Some studies in neonatal care also focus on stress, anxiety and mental health markers in women who gave birth and provide interesting conclusions related to challenges and opportunities in this field of research. The extensive literature review has provided enough information to portray stakeholders’ challenges when adopting IoT technology in pregnancy care. The literature review paves the way for a comprehensive discussion of challenges and opportunities followed by future research direction, which is recapitulated in the subsequent subsection within the purview of this discussion. In our study, we utilize the PRISMA (Preferred Reporting Items for Systematic Reviews and Meta-Analyses) methodology to conduct a comprehensive systematic review of IoT device adoption in maternal healthcare, specifically concentrating on the health monitoring of pregnant women. PRISMA offers a well-structured framework for systematic reviews. Our research questions, a pivotal aspect of PRISMA, steer our examination of the critical determinants affecting IoT device adoption and the challenges within this specialized healthcare context. Our study employs PRISMA to systematically review and synthesize existing literature on the adoption of IoT devices in maternal healthcare. We have formulated research questions that direct our investigation. These research questions include:

- What are the key factors influencing the adoption of IoT devices for maternal healthcare, particularly in the context of monitoring pregnant women’s health?
- What are the primary challenges and barriers faced by stakeholders in implementing IoT devices in maternal healthcare and how do these challenges vary across different demographic and social groups?
- How does prior exposure to technology impact the adoption of IoT devices for health monitoring among pregnant women and what role do education and income levels play in this context?
- What are the implications for privacy, data security and usability in the use of IoT devices for maternal healthcare and how can these issues be addressed to ensure widespread adoption and successful integration?

### 4.4. Analysis of the Literature

In the monitoring of pregnancy health using IoT, researchers have highlighted various security concerns [94]. These include unauthorized access, data breaches, device tampering, data reliability, application security, data accuracy, data integrity, threshold monitoring, alert system security and sensor data validation.

Saarikko et al. [77] emphasize data privacy and recommend robust measures to prevent unauthorized access. Anudeep et al. [78] stress evaluating security through testing to counteract data breaches. El-Aziz and Taloba [47] focuses on protecting against device manipulation. Kalilani et al. [49] highlights securing applications for abnormal readings, advocating for comprehensive security protocols. Cay et al. [55] address data accuracy, proposing sensor validation. De et al. [53] stress safeguarding sensor data integrity and Shakunthala et al. [54] highlight monitoring thresholds.

Govindaraj et al. [56] stress secure alerting mechanisms and Lenka et al. [57] tackle sensor data validation. These findings underscore the need for robust security to protect pregnancy health IoT systems and ensure the well-being of pregnant women and infants. Table 4 shows a summary considering the security protocols and successive risk assessment criteria.

Table 3 provides a comprehensive overview of the home monitoring systems proposed and developed by various authors. We have identified key works that contribute significantly to the discussion of IoT applications in pregnancy monitoring. In the following analysis, we dive into the core contributions of these works to gain a deeper understanding of the role of IoT in maternal health care. Shermi et al. [65] designed an application to improve routine fetal monitoring at home. It covers aspects such as fetal movements and temperature, facilitating timely hospital visits in response to any anomalies Haliima et al. [69] presented a non-intrusive IoT architecture designed for addiction therapy during pregnancy. The system allows remote monitoring and evaluation of pregnant women’s data, as well as providing emergency service contacts for use at home.

Ahmed and Kashem [68] focused on the prediction of maternal risk through a home-based system that used machine learning to assess risk. Venkatasubramanian [71] introduced an IoT-based maternal-fetal health monitoring system, enabling the continuous tracking of health parameters using IoT and AI technologies. Santhi et al. [73] developed a wearable device to continuously monitor health parameters at home, ensuring alerts and data transmission to doctors during emergencies.

Megalingam et al. [74] designed an integrated system for the monitoring of vital parameters and ultrasound screening at home, allowing data storage and transmission. Sato et al. [75] proposed a compact and flexible sensor system for the wireless transmission of vital signs, which enables fetal and maternal monitoring at home using IoT technology. Lyu et al. [76] introduced an Android OS-based multi-communication fusion system for maternal and fetal data monitoring, allowing remote diagnosis and improved accuracy. Saarikko et al. [77] devised a smart wristband and IoT-based monitoring approach to continuously follow health parameters at home during pregnancy.

Table 5 outlines a collection of developed baby care systems and their respective benefits. El-Aziz and Taloba [47] introduced an IoT-based neonatal incubator that offers an environment closely similar to the womb, providing optimal conditions for newborns. de Oliveira Filho et al. [48] designed an IoT-based neonatal incubator with risk management capabilities capable of detecting temperature anomalies to prevent potential risks.

Kalilani et al. [49] developed a cost-effective neonatal incubator unit equipped with heart rate and blood oxygen monitoring, to ensure complete care for newborns. De et al. [53] presented an IoT-based neonatal health monitoring system that continuously tracks vital parameters such as body temperature, acceleration and heart rate.

Cay et al. [55] introduced the Smart Textile Chest Band, a wearable system that monitors respiratory rates and detects apnea in infants through a textile chest band. Singh et al. [44] created an IoT-based neonatal intensive care unit (iNICU) that facilitates continuous monitoring and care for newborns. These innovative systems contribute to advanced baby care, ranging from incubators that simulate womb conditions to wearable devices that improve infant health monitoring.

Table 6 summarizes various studies of the monitoring system and their results. Coulby et al. [43] conducted an exploration of remote healthcare monitoring using accessible IoT technology. Kosma et al. [61] reviewed the impact of new mobile and wearable technologies on the health of pregnant women. Sarhaddi et al. [66] designed and evaluated a long-term IoT-based maternal monitoring system.

Venkatasubramanian [71] developed an ambulatory monitoring system for maternal and fetal health using deep learning and IoT technologies. Lyu et al. [76] created a mobile monitoring system based on multicommunication fusion for maternal and fetal health information. Saarikko et al. [77] conducted a prospective observational feasibility study on continuous IoT-based monitoring of health parameters in pregnant and postpartum women.

Cay et al. [55] introduced the Baby-Guard system, an IoT-based neonatal monitoring solution. Overall, these studies contribute to the growing body of research on healthcare monitoring, showcasing the application of IoT and technology in improving maternal and newborn health.

Table 7 and presents a collection of proposed and developed maternity and neonatal health monitoring devices, each designed to address specific criteria and benefits. Shermi et al. [65] introduced an IoT-integrated maternal-fetal health and labor monitoring System, allowing accurate tracking of fetal movements, temperature, heartbeat and labor discomfort signs. Sarhaddi et al. [66] devised a long-term IoT-based maternal monitoring system, enabling continuous monitoring of physical activity, sleep quality and stress levels to aid early detection of problems. Li et al. [70] proposed a cloud computing and wearable technology-based smart maternal platform, offering comprehensive monitoring of pregnant women’s health. Ahmed and Kashem [68] introduced a risk level prediction system using health data and risk factors for personalized risk assessment. Venkatasubramanian [71] developed an automated deep convolutional generative adversarial network (DCGAN) for continuous monitoring of maternal and fetal health. Other solutions include multi-communication fusion-based user terminal for remote vital sign monitoring system by Lyu et al. [76], cloud-based application enhancing neonatal care system by Singh et al. [46] and sensor-driven neonatal incubator system by El-Aziz and Taloba [47] offering a controlled environment for newborn growth. Collectively, these innovations advance maternal and newborn care through technology-driven monitoring.

Table 8 summarizes various devices/systems and their limitations as identified by different authors. Bjelica et al. [80] introduced an IT ecosystem with concerns about the lack of validation and reliability in questionnaires. Oti et al. [81] developed a Stress Management IoT system but encountered limitations due to a small sample size and a restricted set of stress indicators. Moreira et al. [82] introduced an averaged one-dependence estimator IoT system, but it lacked ethical and privacy discussions. Jara et al. [83] worked on IoT for drug identification, but did not thoroughly investigate scalability. Eswari and Priya [84] focused on the identification of drugs using IoT without evidence of the effectiveness of drug compliance. Beri et al. [85] designed a Smart Health Monitoring System with unclear validity and reliability. Reddy et al. [86] created a remote-controlled IoT system without addressing false alarms or ethical considerations. Alotaibi et al. [87] proposed a Smart Mobile Pregnancy solution with limitations in sample information and implementation challenges. Chen et al. [88] developed an Obstetric Outpatient System but faced limitations in terms of effectiveness and feasibility. Ghimire et al. [89] introduced Prenatal Care IoT with concerns over limited generalizability and data security. Musyoka et al. [90] developed a Smartwatch to monitor blood pressure. However, their findings were limited by a small sample and medical data security issues. Kumar et al. [92] presented an Automated System for PPH Detection. However, their results were hindered by a small sample size, unclear accuracy and a lack of cost-effectiveness analysis.

The use of IoT devices in maternity healthcare confronts a variety of hurdles across demographic groups. Infrastructure and connectivity challenges in rural and disadvantaged urban regions, affordability concerns for low-income people, variable degrees of health literacy and privacy and security concerns, particularly among vulnerable groups, are among them. Cultural, linguistic and access limitations, as well as regulatory and legal difficulties, exacerbate the adoption of IoT devices. In addition, cultural, religious and socioeconomic variables impact their acceptance and there are inequities in access to education and training. To achieve equitable access to maternal healthcare enhanced by the IoT, stakeholders should prioritize affordable and inclusive solutions and community participation [95].

Previous tech exposure influences IoT health monitoring uptake among pregnant women, with familiar users feeling more at ease. Education levels are favorably related to adoption and higher wealth levels allow for easier access to these technologies. Addressing cost and accessibility constraints is critical to fair adoption by people with lower incomes and less tech-savvy. To close the adoption gap, policymakers and healthcare professionals must assure affordability and boost awareness [66].

The adoption of IoT devices in maternal healthcare raises questions about privacy, data security and usability. Robust data security measures, privacy-conscious design, user education and compliance with regulatory frameworks are required to achieve widespread acceptance and effective integration. Transparency, informed consent and user control must be prioritized by stakeholders, who must encourage collaboration between healthcare professionals, technology firms, politicians and pregnant women. These initiatives can help strike a balance between achieving the promise of IoT devices and resolving privacy and security concerns [96].

### 4.5. Challenges and Opportunities in Adoption

Our research is primarily focused on IoT device adoption in maternal healthcare, particularly in the monitoring of pregnant women’s health. Our objective is to address unique challenges and opportunities within this subdomain, providing valuable information to researchers, healthcare professionals and stakeholders. However, we acknowledge that IoT in healthcare has broader applications and challenges beyond our scope of research. Most of the IoT devices used to monitor pregnant women’s activities are biosensors and wearable devices that require placements in the body. Additionally, our comprehensive analysis has identified an overarching concern among stakeholders: fear and concern about radiation exposure on maternal and child health, as indicated in the literature [97]. This fear has become the predominant factor that affects the acceptance and adoption of IoT devices in maternal healthcare. The hesitation to adopt is also due to the irritation of having continuous monitoring devices in the body. Although this hesitation is less in health-conscious women who use IoT devices for running, or athletic activities, it is seen in a higher proportion among other pregnant women. The findings align with the work described in [98], which, in essence, describes that if a person was exposed to technology earlier, it is more likely that the person will adopt new technology. The study supports the findings of the unified theory of acceptance and use of technology (UTAUT) where [99] summarizes that physicians adopting the electronic health record (EHR) system are affected by social influence, facilitation conditions and personal innovation in information technology, which is well in line with our findings, but differs in results related to resistance to change where the article does not find any correlation with what we reported earlier. The level of education also poses a serious challenge to the adoption of IoT. People with more exposure to relevant knowledge quickly adopted the IoT compared to remote areas personnel who initially hesitated. One of the serious questions that kept coming up in the literature was privacy issues related to data. The primary goal of IoT has always been to collect and analyze data to provide better actionable insights. However, researchers consistently agreed that healthcare data is often private information, which is highly sensitive in nature [100]. This challenge forces practitioners to conduct analytical research on private data handling and usable security research. Challenges were also observed with respect to usable data, accuracy, interconnectivity, mobility problems, latency, big data management and analysis, privacy and security and Quality of Service constraints due to time sensitivity, which is a recurring theme in almost all literature and summarized in [39]. The study conducted in Ethiopia [84] concluded that the probability of prenatal care was correlated with adoption barriers such as pregnancy complications, lower educational status of parents, low income, place of residence and zero exposure to the media, which, in summary, can be said that social, economic, personal and educational factors can be considered barriers in the adoption of IoT devices in their lives. A comprehensive point-by-point analysis of the challenges noted in the review is provided in the following.

- Fundamental fear and concern about the impact of radiation from monitoring equipment worn by pregnant women.
- Reluctance to adopt technologies that continuously monitor the body health-conscious appear to experience this discomfort more frequently than health conscious people who started using the device before pregnancy.
- Insufficient knowledge of biosensors and other IoT devices.
- The extremely confidential nature of healthcare data limits the scope of analytical research.
- Barriers to the use of IoT devices at the social, economic, personal and demographic levels.
- Usability, accuracy, connection, mobility, latency, management and analysis of massive data, as well as time-sensitive Quality-of-Service restrictions.

A comprehensive point-by-point analysis of future research implications derived from challenges and discussion is provided below.

- The knowledge, perception, education, age, income and privacy issues pose substantial challenges in adopting IoT devices by stakeholders, which can be future avenues of research.
- Access to internet technology and below-average income are barriers to adoption in low-income countries regardless of stakeholder groups, which is an interesting avenue to explore.
- The argument on data usage and research on private data, while quite prevalent these days, needs to be heavily improved by researchers in IoT, data analytics, data science, machine learning and natural language processing, without which the full potential of IoT cannot be achieved.
- Research on flawless data transmission and usable security needs to be greatly improved to generate trust among all adoption stakeholders.
- Data analysts argued that data generated from IoT devices are often unstructured, unlabeled and ultimately unactionable. Efficient data mining technologies need to be improved in this regard.
- Extensive research is required in the domain of Human-Computer Interaction to improve the usability and user experiences of IoT devices for all stakeholders.
- Improved integration of IoT devices into maternal healthcare settings could be the focus of future research.
- The potential research scope also includes enhancing security and privacy protocols to safeguard the sensitive data of pregnant women and their corresponding healthcare records.
- There is research scope in assessing the development of appropriate regulations and guidelines to control the use of IoT devices in maternity care, ensuring the ethical, safe and secure use of these technologies.

## 5. Conclusions

Pregnant women are prone to mortality and morbidity due to the inadequacy of proper treatment facilities around the world. IoT, mHealth and associated healthcare management processes and systems showed substantial promise by providing a wide array of interconnected devices and monitoring systems to monitor patients and pregnant women in their time of need. This comprehensive literature survey underscores the critical need to improve healthcare care management systems and adopt IoT solutions in the prenatal and neonatal care sectors. Although there is substantial promise in the potential of interconnected devices and monitoring systems to improve the well-being of pregnant women, many challenges and research gaps persist. To address these challenges and pave the way for the adoption of quality devices, new techniques, methods and algorithms are required. These improvements will not only benefit pregnant women, but will also provide valuable insights to the medical community and healthcare professionals. We acknowledge that there is room for further research and innovation in this field and we hope this survey serves as a clear guide for researchers, offering them a well-defined direction to save time and effort while contributing to the improvement of pregnancy care management.

## Figures and Tables

**Table 1:** Summarizing review papers and surveys related to IoT’s role in pregnancy through quantifiable qualitative information.

Source	Year	Publication Type	Limitation
Ahmed et al. [16]	2020	Survey on medical parameters in the remote area	Lacking address of external issues, such as access to healthcare facilities, cultural practices and socioeconomic conditions in the target group.
Ettiyan et al. [58]	2020	Survey on methods and models	Lacking of clarity and a distinct framework
Ahmed et al. [62]	2022	General Survey on methods, results and drawbacks	Lacking detailed technical details on the sensors used, the IoT architecture or how data are processed and communicated.

**Table 2:** Summary of research works that deal with the role of IoT in prenatal care.

Author	Method & Tools	Key Points
Priyanka et al. [63]	Ultrasound scanning method	Developed a smartphone application to automatically track a pregnant woman’s health and fetus activity.
Shermi et al. [65]	Sensor and micro-controller-based methods	Mobile application for fetus’s movements, temperature and heartbeat.
Sarhaddi et al. [66]	Questionnaires and short-term data collection methods	Provides maternal health monitoring throughout the pregnancy and postpartum stages.
Haliima et al. [69]	Proof-of-concept prototype	Device to monitor, gather and evaluate crucial data from pregnant women.
Li et al. [70]	IoT technology and cloud computing	increase the level of obstetric care.
Ahmed and Kashem [68]	Analytical tools and machine learning algorithms	Monitoring and forecasting of the risk level of pregnant women in the context of Bangladesh.
Venkatasubramanian [71]	Linear and non-linear methods for measurement	Various clinical indicators such as MF heart rate, oxygen saturation, blood pressure and uterine tons of expectant are continuously monitored.
Gopalakrishnan et al. [72]	Mapping, bundling and mash-ups Method	IoT-based solution that monitors the vital signs of a pregnant woman and her fetus.
Santhi et al. [73]	Decision Tree classification algorithm and CC3200	A wearable device that continuously logs data while monitoring the critical parameters that need to be tracked for a patient.
Megalingam et al. [74]	Ultrasound scanning method	monitors heart rate, blood oxygen and blood pressure.
Sato et al. [75]	Wireless sensor network	Measures the ECG signal that is comfortably attached to the abdominal area of the mother.
Lyu et al. [76]	Multi-communication fusion-based and wavelet techniques	Pregnant women can monitor maternal and fetal information anywhere they want.
Saarikko et al. [77]	Observational feasibility method	monitoring of health parameters (physical activity, sleep and heart rate) of nulliparous women throughout pregnancy.
Anudeep et al. [78]	RSA algorithm	Incorporates monitoring devices with good data security at a lower cost.

**Table 3:** Summary of research works including methods, applications and pertinent features that deal with the role of IoT in Neonatal care.

Author	Methods	Application	Features
Singh et al. [44]	Deep machine learning Softlayer-based cloud computing infrastructure	integrated neonatology intensive care unit	record data. Vital signs in real-time.
Gadekar and Vaze [45]	logistic regression model	Neonatal Health Monitoring System	Take care of babies. Alarm.
Singh et al. [46]	Unique data transmission protocols and communication interfaces	NEO	Transmit real time data. Automate vital signs.
El-Aziz and Taloba [47]	Arduino UNO microcontroller application programming interface	Neonatal incubator	Take care of babies. Vital signs such as the pulse rate, temperature, humidity and air in the stomach or intestines of the baby.
de Oliveira Filho et al. [48]	IR non-invasive monitoring	Incubator monitoring system	captured thermal image. Monitor temperature
Kalilani et al. [49]	Blynk IoT Platform	Newborn incubator	Body Temperature Measurement, Ambient Temperature Control and Humidity Control.
Kshirsgar et al. [50]	DHT11 framework	neonatal incubator	Temperature control track humidity.
Nivetha and Kumar [51]	Microcontroller and sensor	Intensive care incubator	Heartbeat data. Cry alarm. Humidity sensor.
Cay et al. [52]	Signal processing algorithms, MQTT-IoT based networking	NeoWear	Breathing Rate Track Movements.
De et al. [53]	MCC and WSN	SMCC	Neonatal body temperature. Track movement. Heart rate.
Shakunthala et al. [54]	IoT and sensor modules	Incubator	SMS service. Alarm. Monitoring by sensor.
Cay et al. [55]	MQTT networking architecture and signal processing algorithms.	IMU-Baby-Guard	Pressure sensor pad. Monitor newborns.
Govindaraj et al. [56]	IoT and sensor modules	Incubator monitoring system	Alarm system. Monitor newborn babies.
Lenka et al. [57]	Cloud, IoT and sensor modules	Neonate monitoring system	temperature, heart rate crying sound data

**Table 4:** Table enlisting the systems security protocols and successive risk assessment criteria.

Author	Security & Threat	Risk Assessment
Saarikko et al. [77]	Data Privacy: Unauthorized access to patient health data.	Assess security measures and mitigate data breach risks.
Anudeep et al. [78]	Data Breach: Unauthorized access to sensitive health data from babies and pregnant women.	Regularly assess security measures, conduct penetration testing and apply security patches promptly to mitigate data breach risks.
El-Aziz and Taloba [47]	Device Tampering: Prevent unauthorized modification or tampering with the incubator settings.	Implement physical security measures, such as tamper-evident seals, to protect the device from tampering.
de Oliveira Filho et al. [48]	Device Reliability: Ensure accurate temperature readings for timely response to anomalies.	Regularly calibrate and maintain the sensor hardware to ensure accurate readings.
Kalilani et al. [49]	Application Security: Securing the application that alerts users to abnormal readings.	Implement secure coding practices and regular security assessments for the application.
Cay et al. [52]	Data Accuracy: Ensure accurate tracking of physiological parameters.	Regularly validate signal processing algorithms and sensor accuracy to maintain data accuracy.
De et al. [53]	Data Integrity: Ensuring the accuracy and integrity of sensor data.	Implement data integrity checks and validation mechanisms to detect errors or tampering.
Shakunthala et al. [54]	Data Threshold Monitoring: Monitoring vital metrics for threshold breaches.	Implement continuous monitoring and alerting mechanisms to notify medical professionals of threshold breaches.
Govindaraj et al. [56]	Alert System Security: Ensuring secure and reliable alerting of stakeholders.	Implement secure communication protocols for alert notifications and access controls for alert management.
Lenka et al. [57]	Sensor Data Validation: Ensuring accurate collection and interpretation of sensor data.	Implement validation mechanisms to verify the accuracy of collected sensor data.

**Table 5:** Table enlisting proposed systems and devices related to the infant care system (Neonate).

Author	Developed Baby Care System	Benefits
El-Aziz and Taloba [47]	IoT-Based Neonatal Incubator	Provides optimum conditions like the womb environment
de Oliveira Filho et al. [48]	IoT-Based Neonatal Incubator with Risk Management	Detection of temperature anomalies to prevent risk situations.
Kalilani et al. [49]	IoT-Based Neonatal Incubator Unit	Low-cost neonatal incubator with heart rate and blood oxygen monitoring.
De et al. [53]	IoT-Based Neonatal Health Monitoring System	Continuous tracking of neonatal body temperature, acceleration and heart rate.
Cay et al. [55]	Baby-Guard Smart Textile Chest Band	Monitoring of respiratory rates and apnea detection through wearable system
Singh et al. [44]	IoT-Based Neonatal Intensive Care Unit (iNICU)	Continuous monitoring and data recording.

**Table 6:** Table enlisting proposed systems and devices related to Maternity home monitoring schemes.

Author	Proposed & Developed System	Result and Home Monitoring
Priyanka et al. [63]	Smartphone app for pregnancy monitoring using IoT	Automatic tracking of health and fetal activity at home alerts for abnormal traits.
Shermi et al. [65]	Application for monitoring fetal movements, temperature, etc.	Enhanced routine fetal monitoring at home, helps in timely hospital visits.
Haliima et al. [69]	Non-intrusive IoT architecture for addiction therapy	Remote monitoring and evaluation of pregnant women’s data, emergency services contact at home.
Ahmed and Kashem [68]	System for maternal risk prediction using machine learning	Risk assessment for pregnant women at home using machine learning algorithms.
Venkatasubramanian [71]	IoT-based maternal-fetal health monitoring system	Continuous tracking of maternal and fetal health parameters at home using IoT and AI.
Santhi et al. [73]	Wearable device for continuous parameter monitoring	Continuous health parameter monitoring at home, alerts and data transfer to physicians in emergencies.
Megalingam et al. [74]	Integrated system for ultrasound screening and vital parameters	Monitoring vital parameters and ultrasound screening at home, data storage and transmission.
Sato et al. [75]	Compact, flexible sensor system for fetal and maternal monitoring	Wireless transmission of vital signs at home using a flexible sensor system and IoT technology.
Lyu et al. [76]	Android OS-based multi-communication fusion system	Maternal and fetal data monitoring at home with remote diagnosis, improved precision.
Saarikko et al. [77]	Smart wristband and IoT-based monitoring for health parameters	Continuous monitoring of health parameters at home during pregnancy using a smart wristband.

**Table 7:** Table enlisting proposed systems and developed devices related to Maternity care coordination.

Author	Criteria	Device & System	Benefit
Shermi et al. [65]	IoT Integration	Maternal-Fetal Health and Labor Monitoring System.	The application allows accurate monitoring of fetal movements, temperature, heartbeat and labor discomfort signs
Sarhaddi et al. [66]	IoT-based Systems	Long-Term IoT-Based Maternal Monitoring System.	Continuous monitoring of physical activity, sleep quality and stress levels can help in the early detection of potential health issues
Li et al. [70]	Cloud Computing and Wearable Technology	IoT-based Smart Maternal Platform	comprehensive monitoring of pregnant women’s health.
Ahmed and Kashem [68]	Risk Monitoring and Forecasting	Risk Level Prediction System	personalized risk assessment for pregnant women based on their health data and risk factors.
Venkatasubramanian [71]	Deep Convolutional Generative Adversarial Network (DCGAN)	forecast and control the monitoring of Maternal and Fetal Health (MFH)	The automated system enables continuous monitoring of Maternal and Fetal Health
Lyu et al. [76]	Multi-Communication Fusion-Based User Terminal	multi-communication fusion-based mobile vital signs monitoring s	women can remotely monitor maternal and fetal data from anywhere, enhancing convenience and enabling them to stay informed about their health status.
Anudeep et al. [78]	Effective Communication for Protection	Incorporates monitoring devices	The integration of monitoring devices with good data security measures ensures that sensitive health data remains protected, maintaining patient privacy.
Singh et al. [44]	Cloud-Based Application	Neo-natal intensive care unit (iNICU)	the iNICU system contributes to enhanced care for critically ill neonates by integrating essential calculators and advanced technology.
Singh et al. [46]	Display Interface Module	NEO	The device’s ability to integrate with various devices and sensors ensures comprehensive data collection, providing a holistic view of the newborn’s health status.
El-Aziz and Taloba [47]	Sensor-Driven System	Neo-natal incubator	The neonatal incubator provides a controlled and safe environment that supports the newborn’s growth and development during the crucial early stages of life.
Cay et al. [52]	MQTT-based IoT networking	NeoWear	Movement monitoring, pressure tracking and breathing count are the main features of their device.
de Oliveira Filho et al. [48]	Mobile Cloud Computing (MCC)	Wireless sensor network (WSN)	WSN system allows for continuous monitoring of neonatal health parameters, enhancing the ability to detect and respond to changes promptly.
Cay et al. [55]	Wearable Embedded System	Baby-Guard	The wearable system provides real-time monitoring capabilities, enabling healthcare providers to receive immediate information about neonatal breathing patterns.

**Table 8:** Table enlisting proposed systems and developed devices related to Maternity care coordination alongside their limitations.

Author	Device/System Name	Limitation
Bjelica et al. [80]	IT environment	Lack of validation and reliability in a questionnaire.
Oti et al. [81]	Stress Management IoT	Small sample size, limited stress indicators.
Moreira et al. [82]	Averaged One-Dependence estimator IoT	No ethical/privacy discussion.
Jara et al. [83]	IoT for Medication identification	Lack of scalability investigation.
Eswari and Priya [84]	Drug Identification IoT	No evidence of drug compliance effectiveness.
Beri et al. [85]	Smart Health Monitoring	Unclear validity and reliability.
Reddy et al. [86]	Remote Controlled IoT	No false alarm/ethical discussion.
Alotaibi et al. [87]	Smart Mobile Pregnancy	Lack of sample info, implementation challenges.
Chen et al. [88]	Obstetric Outpatient	Limited by effectiveness and feasibility.
Ghimire et al. [89]	Prenatal Care IoT	Limited generalizability, data security.
Musyoka et al. [90]	Smartwatch for Blood pressure monitoring	Small sample, medical data security.
Kumar et al. [92]	Automated System for PPH Detection	Small sample, unclear accuracy, no cost-benefit.

## Data Availability

This manuscript does not contain additional data.
